# Small RNA Sequencing Based Identification of MiRNAs in *Daphnia magna*

**DOI:** 10.1371/journal.pone.0137617

**Published:** 2015-09-14

**Authors:** Ercan Selçuk Ünlü, Donna M. Gordon, Murat Telli

**Affiliations:** 1 Abant İzzet Baysal University, Faculty of Art and Science, Department of Chemistry, Bolu, Turkey; 2 Mississippi State University, Department of Biological Sciences, Mississippi State, Starkville, Mississippi, United States of America; 3 Abant İzzet Baysal University, Faculty of Art and Science, Department of Biology, Bolu, Turkey; University of Balochistan, PAKISTAN

## Abstract

Small RNA molecules are short, non-coding RNAs identified for their crucial role in post-transcriptional regulation. A well-studied example includes miRNAs (microRNAs) which have been identified in several model organisms including the freshwater flea and planktonic crustacean *Daphnia*. A model for epigenetic-based studies with an available genome database, the identification of miRNAs and their potential role in regulating *Daphnia* gene expression has only recently garnered interest. Computational-based work using *Daphnia pulex*, has indicated the existence of 45 miRNAs, 14 of which have been experimentally verified. To extend this study, we took a sequencing approach towards identifying miRNAs present in a small RNA library isolated from *Daphnia magna*. Using Perl codes designed for comparative genomic analysis, 815,699 reads were obtained from 4 million raw reads and run against a database file of known miRNA sequences. Using this approach, we have identified 205 putative mature miRNA sequences belonging to 188 distinct miRNA families. Data from this study provides critical information necessary to begin an investigation into a role for these transcripts in the epigenetic regulation of *Daphnia magna*.

## Introduction

The discovery of regulatory functions for microRNA molecules has revolutionized gene expression studies, especially in the field of epigenetics. As small non-coding RNAs, miRNAs are approximately 22 nucleotides in length and work as post-transcriptional interfering agents primarily to control mRNA translation and degradation [1]. miRNAs are initially generated by RNA polymerase II activity as ~200-nucleotide long primary miRNA transcripts (pri-miRNA) that fold to generate an imperfect hairpin structure. Pri-miRNAs are subsequently cleaved to generate 70-nucleotide long precursor miRNAs (pre-miRNA) that are exported to the cytosol. In the cytosol, pre-miRNAs are processed by the RNAse III enzyme, Dicer, to generate ~22 nucleotide double-stranded miRNAs. One of the two strands of the miRNA will be assemble with the RNA-induced silencing complex, thereby defining the mRNA target. Recently, miRNAs have come under intensive focus due to their relative abundance, their diversity, and their association with a wide range of cellular mechanisms. For example, miRNAs have been identified in mammals, plants, worms and insects and have been shown to be involved in regulating development, cellular differentiation, proliferation, and death [2]. However, it is their potential involvement in cancer onset and progression that has garnered the attention of many miRNA researchers (reviewed in [2, 3, 4]). To date, many of miRNA studies have focused on a small group of model organisms including mouse, *C*. *elegans*, and *A*. *thaliana* with little effort placed on other sytems. Although interest in the identification of miRNA in non-model organisms has increased in recent years (e.g. *Meloidogyne incognita* [5], *Apostichopus japonicas* [6], *Paulownia fortune* [7]) dissecting the roles of miRNAs with an evolutionary perspective requires additional studies focused on a variety of suitable model organism from different fields.

Recently, *Daphnia* has emerged as a promising model organism with regards to epigenetic studies because of the availability of large genomic and ecological database sets and its unique biological attributes. *Daphnia* species are cyclic parthenogenetic with sex determination between clonal (parthenogenetic) or sexual reproduction controlled by environmental cues [8]. They exhibit morphological alterations such as helmet and neck teeth development within one generation in the presence of predator stress [9]. Detection of predator cues may also lead to the behavioral alteration called diurnal vertical migration (DVM). Characteristic of DVM, *Daphnia* will migrate into the dark and cold hypolimnetic layer of a lake during the day, staying in these unfavorable conditions (food scarcity and low temperature) to avoid fish predation [10]. Clonally reproduce *Daphnia* populations are genetically identical but still show plasticity in behavioral, morphological, and physiological traits. Furthermore, a short life cycle, ease of cultivating under laboratory conditions, and recently described genome sequence data make *Daphnia* an ideal candidate to investigate epigenetic regulation across environmental conditions [11]. In addition, the recent development of targeted gene knockout techniques in *Daphnia* may provide an unparalleled opportunity to investigate the epigenetic role of miRNA within these cells [12].

Despite these advantages, there is an absence in the availability of miRNA sequence data for *Daphnia*. According to the miRBase, there are only 50-conserved miRNA molecules belonging to *D*. *pulex* that have been identified by computational genomics [13]. Inevitably, additional miRNA sequence data will be required to understand and elucidate miRNA mediated molecular regulatory mechanism in this organism. Bioinformatics is a useful approach to identify miRNA sequences from diverse groups of organisms. The identification of both conserved and species-specific miRNAs has primarily relied on computational tools that are homolog-based and utilize sequence conservation or are based on structural and thermodynamic prediction of pre-miRNA hairpin formation. Recently, additional tools have been developed for use in miRNA identification in cancer [14], plant [15], human [16], and mouse [17] systems. However, in all of these cases, sequence information of the target organism is required [14]. Unfortunately, genomic information for *D*. *magna* is limited, making most of these available tools not applicable. Thus in this study we aimed to use modified Perl codes to identify conserved miRNA sequences within a *D*. *magna* small RNA library using *D*. *pulex* genome information.

## Materials and Methods

### *D*. *magna* culturing and total RNA isolation

*D*. *magna* strains were purchased from MBL Aquaculture and maintained in 2 L glass beakers filled with oxygenated, deionized water kept at ambient temperature (20°C) in a light controlled room (16h light and 8 h dark) for two weeks before RNA isolation. *D*. *magna* were fed daily with freshwater algae (*Pseudokirchneriella subcapitata*; 3.5 x 10^6^ cells/mL) and YCT (yeast, cereal leaves, tetramin) mixtures at 7mL and 10mL per L of water, respectively.

Total RNA <200 nucleotides in length was extracted from 5 whole-body *D*. *magna* adults randomly selected from culture. Total RNA isolation was performed using mirVana miRNA Isolation Kit according to the manufacturer’s instructions (Life Technologies). Isolated nucleic acid was quantitated spectrophotometrically, and the integrity of the RNA checked by electrophoresis through a 15% acrylamide (19:1 acrylamide:bis acrylamide), 8M Urea, 1X TBE 0.75mm vertical gel followed by ethidium bromide staining.

### Small RNA sequencing

Total small RNA samples were submitted to HudsonAlpha Institute for Biotechnology (Huntsville, Alabama) for quantitation, small RNA library preparation, and sequencing using the Illumina HiSeq 2000 next generation sequencing platform. Data for four million 50 base pair, single-end reads were analyzed for miRNA signatures by computational sequence analysis.

### Computational sequence analysis

Small RNA library sequence data was processed using designed Perl codes (available upon request) to remove redundant sequences. A simple Perl code was also generated to blast small RNA sequences against a database that is compatible with Bioperl tool kit [18]. A total of 30,424 known mature miRNA sequences belonging to 203 different species was downloaded in FASTA format via FTP from the miRBase miRNA database (ftp://mirbase.org/pub/mirbase/CURRENT/mature.fa.gz) [19]. Given the short sequences, the code selected sequences having more than 90% identity to reduce the risk of false positives.

For secondary structure prediction studies, a Perl code was written to identify miRNA precursor sequences. The code searched for putative mature miRNA sequences in *D*. *pulex* chromosomal sequences (downloaded from NCBI) and extracted the matching sequence in addition to 80 nucleotides upstream and downstream of the reference miRNA. miRNA precursor sequences obtained in this manner were analyzed using the RNA Folding Form application at http://mfold.rna.albany.edu [20] to predict secondary structure using the default software settings. Structural output files in Vienna format were uploaded to the Mfold server using Structure Display and Free Energy Determination application for structure display [21].

## Results and Discussion

### Small RNA sequencing

**Fig 1:**
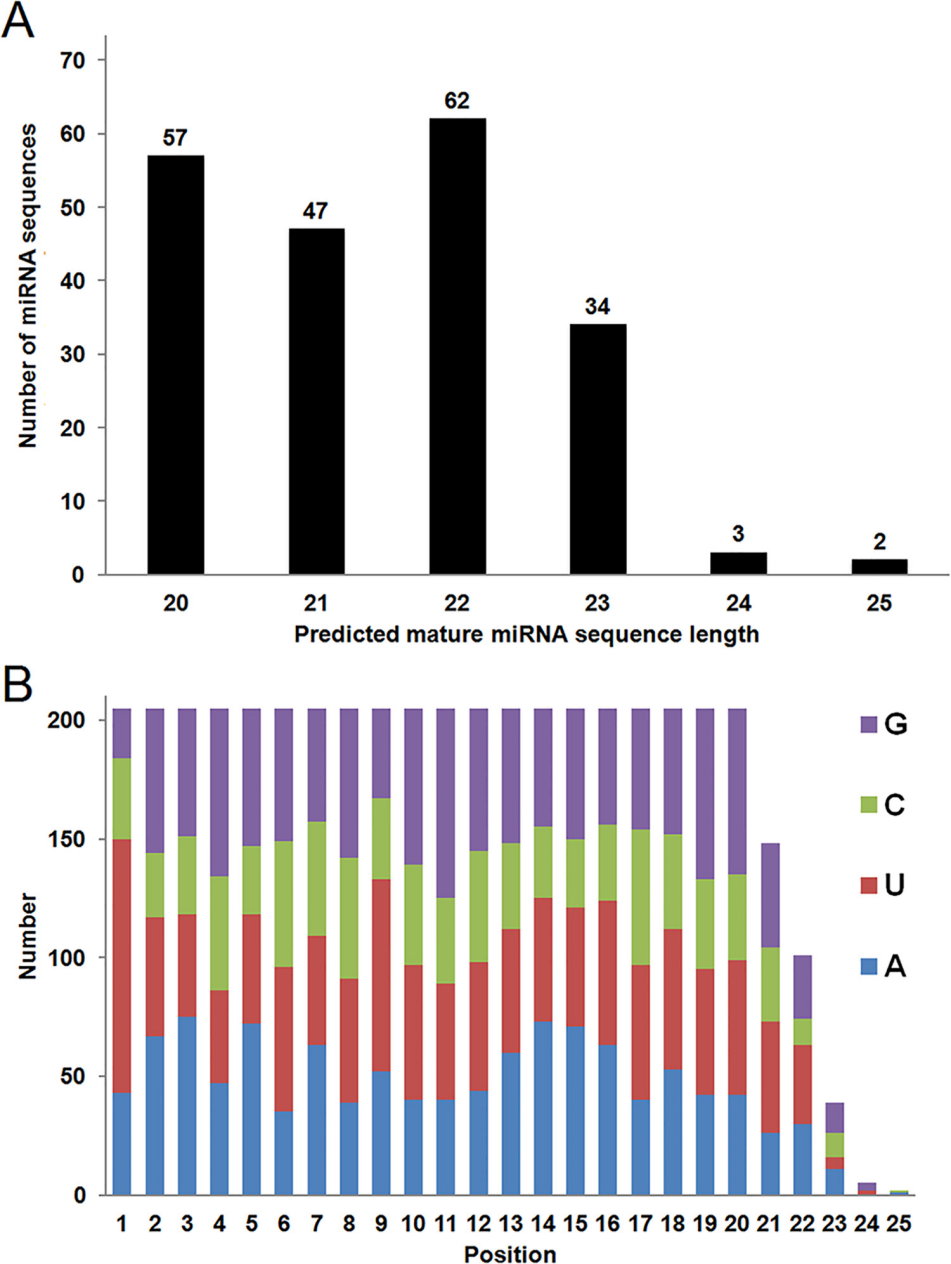
Length and base distribution of identified putative mature miRNA sequences. (A) Nucleotide lengths of each identified miRNA sequences were analyzed. Lengths from 20 nucleotides to 25 nucleotides were identified and the total number of predicted miRNA sequences for each corresponding sequence length is indicated. (B) Base distribution at each position of the 205 identified miRNA sequences are indicated.

*D*. *magna* small RNA (less than 50 nucleotide) library were sequenced using the Illumina sequencing platform. Approximately 4 million raw reads obtained from sequencing were processed by Perl codes to remove multiple reads, contaminants, and adaptor sequences. The resulting data file contained 815,699 sequences which were run in encoded Perl blast code against a database file containing all known miRNA sequences obtained from the NCBI database and formatted using Blastdb tool. Considering the short length of miRNA sequences, we only parsed the data having 90% or higher identity and higher than 0.1 E value. The analysis revealed 205 putative mature miRNA sequences belonging to 188 miRNA families (detailed data analysis is given in S1 and S**2** Tables). As expected, the read length of 22 nucleotides was the most abundant at 30.2% of the total miRNAs identified. This was followed by reads with lengths of 20 nucleotids (27.8%) and 21 nucleotides (22.9%), respectively (Fig 1A). Base distribution analysis identified a strong preference for a purine at the first position of the mature miRNA, with 107 of the transcripts having a uracil at the first position and 43 starting with a adenine (Fig 1B).

### Conservation of putative *D*. *magna* miRNAs

Conservation of proposed *D*. *magna* miRNA sequences among other species is an important criteria for the initial validation of miRNA transcripts. Based on a cutoff of 90% sequence identify, the data set had the highest number of conserved miRNA transcripts when compared to human miRNAs (105 hits), which was followed by *M*. *musculus* (90 hits), *D*. *melanogaster* (45 hits), *D*. *pulex* (41 hits), and *C*. *elegans* (14 hits) miRNAs, respectively (Table 1 and S3 Table). Given that these numbers are dependent on the number of miRNA identified for each species, the data presented in Table 1 does not represent a homological comparison among species. However, the data does suggest that *D*. *magna* is a suitable model organism for the study of miRNA functions.

**Table 1:** Conservation of putative *D*. *magna* miRNAs among model species.

Species	Number of predicted miRNAs	Number conserved with *D*. *magna*	% Conserved with *D*. *magna* *
*Homo sapiens*	2578	105	4.1
*Mus musculus*	1908	90	4.7
*Drosophila melanogaster*	426	45	10.6
*Daphnia pulex*	45	41	91.1
*Caenorhabditis elegans*	368	14	3.8

Each sequence was compared for its existence in human and four model species (*D*. *pulex*, *M*. *musculus*, *D*. *melanogaster*, *C*. *elegans*). Total number of each predicted sequence hit for each species were counted. In addition, total number of conserved miRNAs with *D*. *magna* were divided to total identified number miRNAs for each species were calculated. The expansion of Table 1 including the analysis for each species in the database is given as S3 Table.

* Number conserved with *D*. *magna /*Total number of predicted miRNAs

### Validation of *D*. *magna* miRNAs by secondary structure prediction

**Fig 2:**
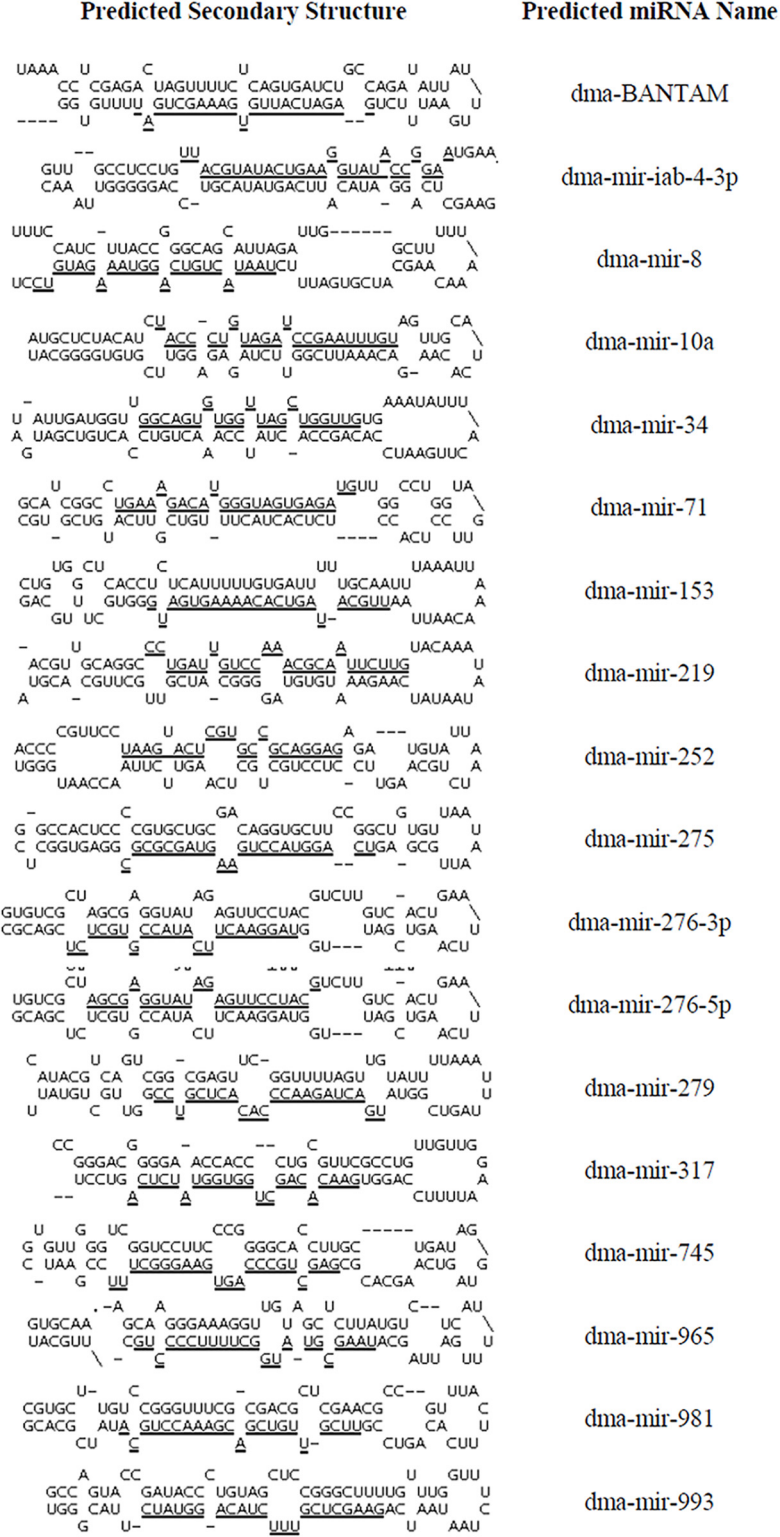
Predicted secondary structure visualizations for putative *D*. *magna* miRNA sequences. The RNA Folding Form application in Mfold server was used to determine the secondary structure of precursor microRNA sequences obtained by parsing the nucleotide sequences selecting an 80 nucleotides upstream and downstream window. Default software settings were used for structural analysis and data downloaded in Vienna file format. To illustrate secondary structures, Vienna formatted data files were used in Structure Display and Free Energy Determination applications in Mfold server. For the ease of identification, the corresponding predicted miRNA sequences are underlined.

Since *D*. *magna* chromosome sequence data is not currently available, *D*. *pulex* chromosomal scaffold sequence data obtained from the NCBI database was used as a chromosomal reference for the identification of miRNA precursor information. A simple Perl code was developed to extract precursor miRNA data from *D*. *pulex* chromosomal sequence for putative miRNAs that showed 100 percent homology with *D*. *pulex*. Given that 4 of the 41 *D*. *magna* miRNAs showed less than 100% homology with *D*. *pulex*, only 37 of the miRNAs were analyzed for secondary structure predictions. 5185 scaffold sequences, with sequence lengths ranging from 1,000 to 3,777,634 nucleotides, were searched for positive matches to the 37 *D*. *magna* putative mature miRNAs. Setting the matching region as the core, sequence from the region ~80 nucleotides upstream of the start of the mature miRNA and ~80 nucleotides downstream of the miRNA was selected (S4 Table). Extracted miRNA precursor sequences identified in this manner were analyzed using Mfold software to predict sequence secondary structures. In total, upstream and downstream nucleotide sequence information was obtained for 18 of the 37 *D*. *pulex* homologs and is available in S3 Table. All 18 fit parameters characteristic of microRNAs in that they: a) were predicted to form irregular stem-loop structures with the mature miRNA located on one of the two arms of the hairpin (Fig 2) and b) had a limited number of mismatches (including insertions and deletions) between the mature miRNA and its complementary passenger strand, with 4 and 5 being the most common number of mismatches (found in 11 of the 18 predicted miRNAs). The inability to determine secondary structure for the remaining 19 *D*. *magna* miRNAs was likely due to the incomplete and fragmented nature of the *D*. *pulex* scaffold sequences used for analysis.

Since *Daphnia* is a promising model for development-based studies, we determined the number of predicted *D*. *magna* miRNAs that are likely to be involved in regulating various developmentally linked signaling pathways by comparing them to miRNAs known to be implicated in human (total of 145 miRNAs) and *Mus musculus* (total of 52 miRNAs) development (Table 2). We specifically focused on calcium, Hedgehog, WNT, Notch and chemokine signaling pathways in *Homo sapiens* and *Mus musculus*. Using data downloaded from TarBase [22], these miRNAs were compared against the 105 predicted miRNAs conserved with *Homo sapiens* and 90 predicted miRNAs conserved with *Mus musculus* (S5 Table). In total, 52 of the 205 miRNAs identified in *D*. *magna* are predicted to have a role in regulating cellular signaling cascades implicated in controlling developmental pathways.

**Table 2:** Conservation of predicted miRNAs involved in development-related signaling pathways.

Organism	Signaling pathway	Number of conserved miRNAs with *D*. *magna*	Total number of known miRNAs	% Conserved with *D*. *magna* *
*Homo sapiens*	Calcium	18	48	37.50
	Hedgehog	23	45	51.11
	WNT	41	104	39.42
	Notch	24	49	48.98
	Chemokine	37	88	42.05
	*Total*	52	145**	35.86
	Calcium	11	36	30.56
	Hedgehog	11	24	45.83
*Mus musculus*	WNT	11	36	30.56
	Notch	7	16	43.75
	Chemokine	12	34	35.29
	*Total*	18	52**	34.62

*Number of conserved miRNAs with *D*. *magna* /Total number of signal pathway miRNAs.

**Total number is achieved after removal of overlapping miRNAs among signaling pathways.

Comparing putative *D*. *magna* miRNAs with homologs reported for *Homo sapiens*, *Mus musculus*, *D*. *melanogaster*, and *C*. *elegans* found that 55% of these miRNAs (105 of 188) were present in one or more of the four model organisms (S6 Table). Homologs for 13 of these 105 have only been reported for humans, 36 of the *D*. *magna* miRNAs have family members in humans and mouse but not *D*. *melanogaster* or *C*. *elegans*, and 2 miRNA family members were found in all 5 organisms (*miR-124* and *let-7*).

## Conclusion

We have identified 205 putative miRNA sequences expressed in *D*. *manga*. These are the first and the largest number of miRNA sequences directly isolated, and identified, by a comparative genomics approach in *D*. *manga*. These sequences included 41of the 45 miRNA sequences previously predicted for *D*. *pulex* and available from the MirBase database, confirming the strength of this approach. The identification of these sequences has greatly improved the miRNA database for *Daphnia* and research efforts focusing on epigenetic regulation in this model organism.

Identification of putative *D*. *magna* miRNAs were based on sequence conservation with known miRNAs, therefore it is likely that species-specific miRNAs were overlooked in our analysis and the 205 miRNAs identified in this study is an underestimate of the miRNAs present in *D*. *magna*. Given that many of the bioinformatics tools currently used to identify miRNAs rely of the availability of genome sequence information, the identification of additional miRNAs for this model organism will likely have to await the availability of a more complete genome data set.

When compared to human and model organisms, microRNA families identified in this study validate potential for using *D*. *magna* as a model for miRNAs based studies. As presented in S6 Table, there are several miRNA families conserved between human and *D*. *magna* for which no members have been identified in other model organisms such as mouse, *C*. *elegans* or *D*. *melanogaster*. Many of the miRNAs were identified by RNA sequencing of normal versus cancerous cells and differentiated versus undifferentiated stem cells [23, 24]. Results from additional studies have suggested a role for these miRNAs in regulating cell proliferation and differentiation [25, 26]. Given the potential importance of these miRNAs in normal cellular activities, the identification of miRNA homologs in *D*. *magna* may provide a unique opportunity to study the function of these miRNA family members in a more tractable system.

Using a bioinformatics approach and the *D*. *pulex* genome as the template, we were able to confirm that 18 of the 205 putative miRNAs were likely to have pre-miRNA transcripts with secondary structure predictions consistent with the formation of a hairpin, a defining characteristic of miRNAs. Support for the validity of the remaining miRNAs will likely require completion of the *D*. *magna* genome. In addition to microRNAs, small non-coding RNAs also include those of the piRNA family. These transcripts range in size from 26–30 nucleotides in length in *D*. *melanogaster*, but can be as short as 21 nucleotides as in the case of *C*. *elegans* [27]. Unlike miRNAs, these transcripts do not form a double-stranded intermediate, utilize a distinct cellular pathway for maturation, and share little sequence conservation outside of a preference for a uracil at the 5’ position. Although many of the putative miRNAs identified in this study fit several of the descriptors for a piRNA (i.e. 21 nucleotides in length, uracil at position 1 of the mature RNA transcript), the identification of homologs with 90% or higher identity in other species argues against them falling into this category.

Based on sequence conservation with human and mouse, a number of the identified miRNAs are likely to be involved in regulating the expression pattern of genes involved in influencing various developmental pathways (Table 2), information that will provide a useful opportunity to understand and elucidate similar molecular mechanisms in *D*. *magna*. Recently developed gene manipulation techniques during the embryonic development of *Daphnia* [13], and the availability of the related set of common miRNA sequences are likely to provide a significant opportunity to reveal epigenetic regulation of embryonic stages in this model organism.

## Supporting Information

S1 Table Raw data after miRNA sequence analysis. Table represents the raw data that shows the parameter values for each sequence having 90% or higher identity and 0.1 E-value. The raw data was obtained after running the blast code using 815,699 reads that remained after the clean-up process. miRNAs that show homologues in humans and model organisms (*Mus musculus*, *Drosophila melanogaster*,*Caenorhabditis elegans* and *D*. *pulex*) are presented.
(XLSX)

S2 Table Predicted miRNA sequences and corresponding mature miRNA sequences. Predicted miRNA sequences were named by adding a dma- suffix to homologous sequence names identified in other species. Trimmed versions of raw sequence read data are presented as predicted mature miRNA sequence. A simple Perl code was used to trim adaptor sequences from the raw data and calculate the sequence lengths.
(XLSX)

S3 Table Comparison of putative miRNA sequences from *D*. *magna* with available miRNAs. Data is an expansion of that presented in Table 1. The total number of predicted sequence hits for each species was determined. The percent of conserved miRNA transcripts with *D*. *magna* was calculated by dividing the number of homologous miRNAs by the total number of miRNAs identified for each species.
(XLSX)

S4 Table Extracted miRNA precursor sequences from *D*. *pulex* genome. Table represents the raw data for pairwise analysis of miRNA sequences that matched in *Daphnia pulex* genome. Extracted sequence and corresponding scaffold information are represented for the 18 predicted *D*. *magna* miRNAs.
(XLSX)

S5 Table Pairwise comparison of predicted *D*. *magna* miRNAs with those involved in development-related pathways in *Homo sapiens* and *Mus musculus*. Table represents the list of predicted miRNAs involved in calcium, Hedgehog, WNT, Notch and chemokine signaling pathways in *Homo sapiens* and *Mus musculus*. For each signaling pathway, homologous miRNAs matching those downloaded from the TarBase database are presented. ‘Total’ represents the unique number of development specific miRNAs based on their involvement in one of the four signaling cascades: calcium, Hedgehog, WNT, Notch and/or chemokine pathways.
(XLSX)

S6 Table Comparison of miRNA family members between *D*. *magna* and other model species. miRNA families with homologous predicted sequence hits were compared for their presence in *Homo sapiens*, *Mus musculus*, *Drosophila melanogaster*, *and Caenorhabditis elegans*. A predicted miRNA family member found in *D*. *magna* and one of the four model species is indicated with a green “√” sign; a red “X” indicates that a homolog for the putative *D*. *magna* miRNA was not found in that species.
(XLSX)
